# Significance of age and sex in botulinum neurotoxin dosing for adductor spasmodic dysphonia

**DOI:** 10.1002/wjo2.88

**Published:** 2023-01-29

**Authors:** Richard Heyes, Charles H. Adler, Nan Zhang, David G. Lott, Stephen F. Bansberg

**Affiliations:** ^1^ Department of Otorhinolaryngology ‐ Head and Neck Surgery Mayo Clinic Arizona Phoenix Arizona USA; ^2^ Department of Neurology Mayo Clinic Arizona Phoenix Arizona USA; ^3^ Department of Biostatistics and Bioinformatics Mayo Clinic Arizona Phoenix Arizona USA

**Keywords:** adductor, age, gender, outcomes, sex, spasmodic dysphonia

## Abstract

**Objectives:**

This study aims to analyze the impact of age and sex on botulinum neurotoxin (BoNT‐A) dosing and outcomes in adductor spasmodic dysphonia (AdSD).

**Methods:**

A database review of all spasmodic dysphonia patients treated with BoNT from 1989 to 2018 at the Mayo Clinic in Arizona was performed. Only patients who had received ≥4 injections of BoNT‐A for AdSD were included. Patients were divided into two cohorts to analyze age, with an age of first treatment cutoff of 60 years. Patients were divided into male and female cohorts to analyze sex.

**Results:**

The final analysis included 398 patients. The mean dose of BoNT‐A per treatment was significantly higher in the younger cohort (4.4 vs. 3.9 units, *p* = 0.048). The mean maximal benefit was similar (72% vs. 70%, *p* = 0.48); however, the mean length of benefit was significantly shorter in younger patients (3.0 vs. 3.6 months, *p* < 0.01). The mean BoNT‐A dose was significantly higher in the female cohort (4.2 vs. 3.6 units, *p* = 0.02). The mean maximal benefit was similar (69% vs. 75%, *p* = 0.58), as was the mean length of benefit (3.2 vs. 3.5 months, *p* = 0.11).

**Conclusions:**

This study suggests that age and sex influence BoNT‐A dosing and outcomes in AdSD.

## Key Points

- Significant findings of the study: The younger cohort received higher doses of botulinum neurotoxin (BoNT‐A) and experienced a shorter length of benefit, but the maximal benefit achieved was similar to the older cohort. Females received higher doses of BoNT‐A with other outcome metrics similar to males.
- What this study adds: This study represents the most highly powered publication to date addressing the impact of age and sex on the nature and efficacy of BoNT‐A treatment for adductor spasmodic dysphonia. Its findings can assist physicians in patient counselling and BoNT‐A dose selection.

## INTRODUCTION

Spasmodic dysphonia (SD) is a task‐specific focal laryngeal dystonia.^1^ Adductor spasmodic dysphonia (AdSD), characterized by a harsh, strain‐strangled voice with breaks on vowels in speech, is the most common variant of the condition. Vocal tremor co‐occurs in 30%–60% of AdSD patients.^1^, ^2^

Based on the significant symptom response of blepharospasm to intermittent injections of botulinum neurotoxin (BoNT‐A), Blitzer et al. performed the first laryngeal injection of BoNT‐A for SD in 1984.^3^, ^4^ The precise mechanism that BoNT‐A improves SD symptomatology is unknown. Its effect is not fully explained by chemodenervation of the thyroarytenoid muscle since other adductor muscles are relatively unaffected and some patients experience improvement in concurrent extralaryngeal dystonias.^5^ It may therefore result in disease modulation at a central level.^6^ Unlike many pharmacologic agents, the therapeutic BoNT‐A dose cannot simply be calculated according to weight, body mass index, or kidney function; dosing is patient‐specific and tailored to their symptoms and desires. Despite its use in the larynx being considered “off‐label” by the US Food and Drug Administration (FDA), BoNT‐A remains the first‐line treatment for SD and is the recommended primary management strategy in the American Academy of Otolaryngology‐Head and Neck Surgery's Clinical Practice Guideline for dysphonia.^7^, ^8^ There is currently no cure for SD. Thalamic deep brain stimulation may become a treatment option to modulate the central pathophysiology of SD.^9^, ^10^

Limited data are presented in the literature regarding the impact of age and gender on outcomes of BoNT‐A treatment in AdSD. This study aims to analyze the impact of age and sex on outcomes in AdSD treated with BoNT‐A. It was hypothesized that these groups would be similar in baseline characteristics and outcomes with BoNT‐A treatment, with outcome measures including mean BoNT‐A dose, mean self‐perception of maximal benefit, mean perceived length of benefit, mean time between injections, mean breathiness duration, and mean duration of dysphagia to liquids.

## METHODS

A retrospective database review of all SD patients treated with BoNT‐A from 1989 to 2018 at the Mayo Clinic in Arizona (Phoenix) was performed. This study was approved by the Mayo Clinic Institutional Review Board (10‐007839).

All patients were evaluated by an otolaryngologist, a movement disorder neurologist, and a speech‐language pathologist. Evaluation was performed by the same otolaryngologist over the entire study period and the same neurologist over 28 years. Medical history, laryngeal examination, voice evaluation, and screening neurological evaluation were performed on all patients. The patient's medical history and auditory‐perceptual evaluation provided the information to make the diagnosis for most patients. Fiberoptic transnasal laryngoscopy influenced the diagnosis in some cases.

Patients diagnosed with AdSD, with or without vocal tremor, were included in this study. Patients with these diagnoses who also manifested vocal characteristics of muscle tension dysphonia were included. Patients with abductor SD, mixed SD, primary muscle tension dysphonia, isolated vocal tremor, dysarthria, Tourette's syndrome, and other voice disorders were excluded from the analysis.

Only patients who had received ≥4 injections of BoNT‐A were included. Injections are performed using electromyography (EMG) guidance with the subcutaneous injection of local anesthetic. BoNT‐A is injected into each thyroarytenoid muscle using a 37‐mm, 27‐guage Teflon‐coated monopolar EMG injection needle. A 100‐unit vial of BoNT‐A diluted with preservative‐free saline to a concentration of two units per 0.1 ml is prepared for each patient. At our institution, patients are initiated at a dose of 1.2 units bilaterally with subsequent dose titration guided by patient response and preference. Some patients convert to unilateral injection. We elected to exclude the first two injections from the analysis. The third dose received is the first included in the analysis with a subsequent injection required to collect outcome data. To analyze the role of age, patients were divided into two cohorts: those with a first treatment aged 60 or younger and those with a first treatment over the age of 60. Patients who had previously received treatment at another institution were placed in the cohort based on the age of their first outside injection and their first two injections at our institution were excluded. To analyze the role of sex, patients were divided into two cohorts: male and female.

Details recorded include sex, age of symptom onset, the time between first injection and last injection, number of injection sessions, presence of vocal tremor, presence of a coexisting movement disorder, and family history of SD or other dystonias.

Outcomes compared included mean BoNT‐A dose, mean self‐perception of maximal benefit (patients are asked to attribute a percentage to the maximum benefit received from the previous injection), mean perceived length of benefit from the previous injection (in weeks), mean time between injections, mean breathiness duration (patients are asked to provide a duration of breathiness in days or weeks), and mean duration of dysphagia to liquids (patients are asked to provide a duration of dysphagia to liquids in days or weeks). Data were collected at the patient's next injection appointment using a standardized questionnaire that remained unchanged over 30 years. Dose refers to the total amount administered in a treatment session (i.e., the cumulative dose in bilateral injections). Individual patient averages were calculated and the averages of these were analyzed. This was performed to prevent analysis from being skewed by patients who have received many treatments (up to 80). Linear regression was used to adjust for sex or age, coexistent vocal tremor, and other movement disorders. Analysis was conducted using advanced statistical software and a two‐sample *t*‐test or Wilcoxon rank sum test when applicable. A *p*‐value < 0.05 was used to define statistical significance. Means are presented with the standard deviation in Tables 2, 4, and 5, and with the standard error in Table 3. Medians are presented with the interquartile range.

## RESULTS

**Figure 1:**
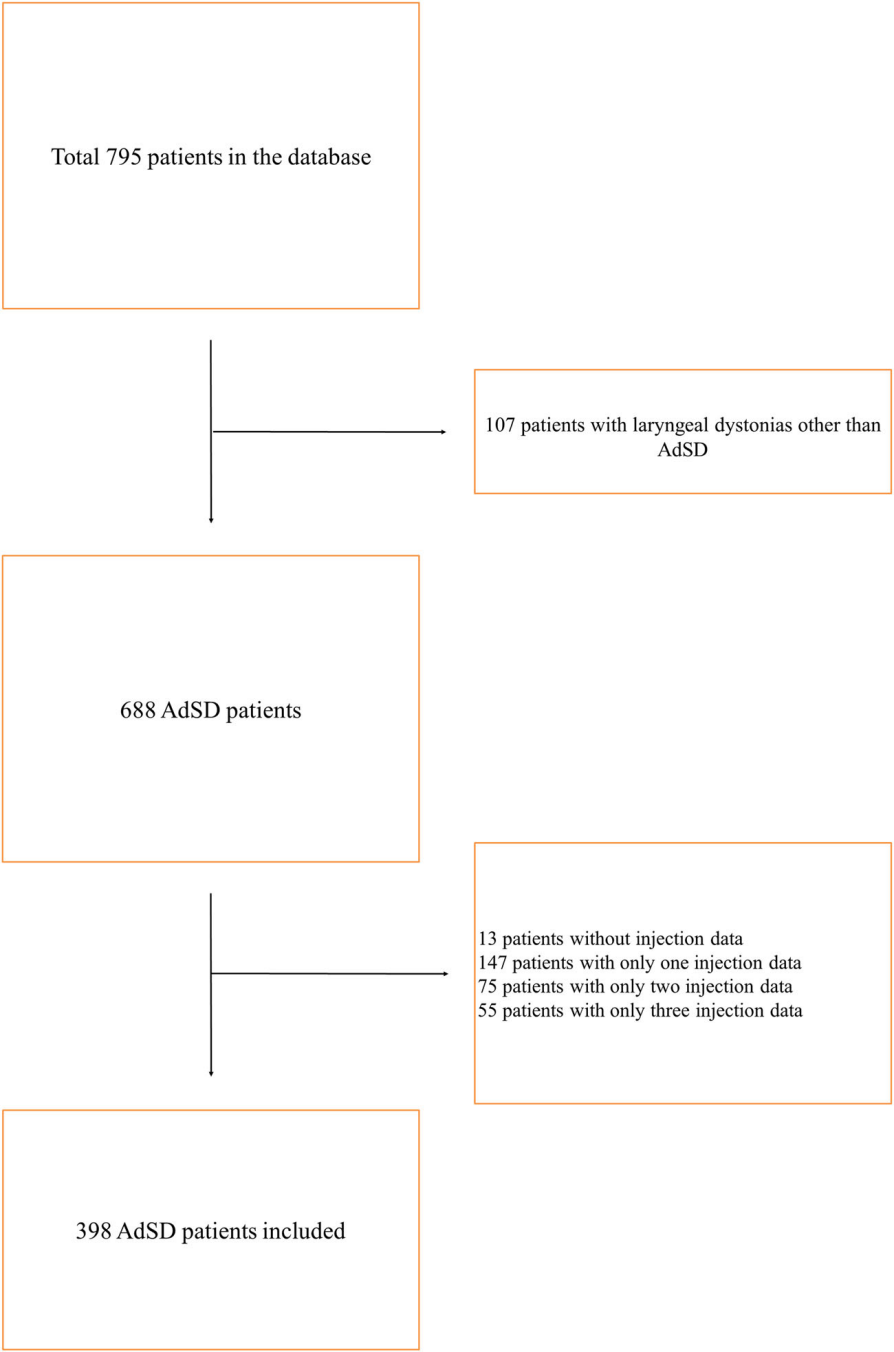
Algorithm displaying the use of the inclusion criteria to generate the cohort of patients studied.

Our database search identified 795 patients. The final analysis included 398 patients (Figure 1).

### Analysis by age

Of the included patients, 199 received their first injection ≤60 years old (50%) and 199 received their first injection after their 61st birthday (50%). Demographic details are displayed in Table 1. Younger patients were more likely to be male than in the aged cohort (27% vs. 12%, *p* < 0.01). The length of follow‐up was similar (median = 6 years) but younger patients received a higher median number of treatments (13 vs. 9, *p* < 0.01). Older patients were more likely to have coexistent vocal tremor (*p* < 0.01) or other movement disorders (*p* = 0.03). There was no difference in the family history of SD or other dystonias. Documentation of age of dysphonia onset was present in slightly more than half of patients, with means of 40 years and 62 years in the younger and older cohorts, respectively. Table 2 displays data relating to the age of dysphonia onset.

**Table 1:** Demographic data for analysis with age as the variable

Item	≤60 years old (*n* = 199)	>60 years old (*n* = 199)	Total	*p*‐value
Female	145 (73.0%)	175 (88.0%)	320 (80.0%)	<0.01
Time from first to last injection (years)	6 [3, 13]	6 [3, 10]	6 [3, 11]	0.26
Number of injections	13 [7, 24]	9 [5, 17]	11 [6, 21]	<0.01
Coexistent vocal tremor	74 (37.0%)	136 (68.0%)	210 (53.0%)	<0.01
Additional movement disorder	21 (11.0%)	37 (19.0%)	58 (15.0%)	0.03
Family history of spasmodic dysphonia	3 (1.5%)	0 (0)	3 (1.0%)	0.24
Family history of tremor	13 (6.5%)	17 (8.5%)	30 (7.5%)	0.57
Family history of another movement disorder	4 (2.0%)	2 (1.0%)	6 (1.5%)	0.69

**Table 2:** Mean age of dysphonia onset and percentage of patients for whom this information was available

Item	≤60 years old (*n* = 199)	>60 years old (*n* = 199)	Total
Data present	110 (55.0%)	119 (60%)	229 (58%)
Mean age at onset (SD)	40 (12)	62.3 (8.6)	50.5 (15.3)

The mean BoNT‐A dose was significantly higher in the younger cohort (4.4 vs. 3.9 units, *p* = 0.048). The mean maximal benefit was similar (72% vs. 70%, *p* = 0.48); however, the mean length of benefit was significantly shorter in younger patients (3.0 vs. 3.6 months, *p* < 0.01). Length of Posttreatment breathiness did not differ, with a mean of 16 days in both cohorts. Length of posttreatment dysphagia to liquids did not statistically significantly differ (3.3 vs. 4.6 days, *p* = 0.09). Table 3 displays these data.

**Table 3:** Dosages and treatment outcomes with age as the variable

Item	≤60 years old (*n* = 199)	>60 years old (*n* = 199)	*p*‐value
Mean dose (Units)	4.4 (0.2)	3.9 (0.2)	0.048
Mean % maximal benefit	71.5 (1.8)	69.6 (1.8)	0.48
Mean duration of benefit (months)	3.0 (0.1)	3.6 (0.1)	<0.01
Mean time between injections (weeks)	37 (3)	39 (3.0)	0.65
Mean breathiness duration (days)	16.0 (0.6)	16.4 (0.6)	0.63
Mean dysphagia duration (days)	3.3 (1.8)	4.6 (0.5)	0.09

### Analysis by sex

Of the included patients, 320 (80%) were female and 78 (20%) were male. Demographic details are displayed in Table 4. The female cohort demonstrated older mean age at the first injection (60 vs. 54 years, *p* < 0.01). Female patients were more likely to exhibit a coexistent vocal tremor (59% vs. 27%, *p* < 0.01). Cohorts were similar regarding the length of follow‐up, the number of treatments, additional movement disorders, and family history. The mean BoNT‐A dose was significantly higher in the female cohort (4.2 vs. 3.6 units, *p* = 0.02). The mean maximal benefit was similar (69% vs. 75% (*p* = 0.58), as was mean length of benefit (3.2 vs. 3.5 months, *p* = 0.11). Length of posttreatment breathiness and posttreatment dysphagia to liquids were similar. Table 5 displays these data.

**Table 4:** Demographic data for analysis with sex as the variable

Item	Female (*n* = 320)	Male (*n* = 78)	Total	*p*‐value
Age at first injection	60 (15)	54 (13)	59 (15)	<0.01
Time from first to last injection (years)	6 [3, 11]	6 [3, 11]	6 [3, 11]	0.90
Number of injections	11 [5, 21]	10.5 [6, 22]	11 [6, 21]	0.85
Coexistent vocal tremor	189 (59%)	21 (27%)	210 (53%)	<0.01

**Table 5:** Dosages and treatment outcomes with sex as the variable

Item	Female (*n* = 320)	Male (*n* = 78)	*p*‐value
Mean dose (Units)	4.2 (2.5)	3.6 (2.1)	0.02
Mean % maximal benefit	69 (25)	75 (20)	0.58
Mean duration of benefit (months)	3.2 (1.8)	3.5 (2.5)	0.11
Mean time between injections (weeks)	37 (39)	40 (47)	0.23
Mean breathiness duration (days)	16.1 (0.6)	16.8 (0.6)	0.47
Mean dysphagia duration (days)	4.0 (6.1)	3.6 (6.8)	0.97

## DISCUSSION

Though widely accepted, BoNT‐A treatment for AdSD is not standardized; because no clear guidelines exist, protocols in BoNT‐A treatment of patients with SD vary among physicians. A survey of practicing laryngologists in the United States found that 70 participants collectively reported treating >4000 patients with SD annually.^7^ Most of these physicians reported performing BoNT‐A treatment exclusively in the office, using EMG alone as guidance, and targeting the thyroarytenoid muscles in AdSD.^7^ A substantial majority prefer to start with bilateral injections over unilateral injections. The most commonly reported starting dosage was 1.25 units per side. Most felt that male and female patients do not require different BoNT‐A dosages for AdSD treatment and the expected interval between injections was 3–4 months. The physicians estimated that 95% of patients treated with BoNT‐A received benefit.^7^

This study aimed to analyze the influence of both age and sex on BoNT‐A dosage and treatment outcomes in AdSD. For the analysis of age, patients were initially divided into three cohorts (<50, 50–60, and >60 years of age at first injection). The demographics, clinical characteristics, and outcomes between patients <50 years old and 50–60 years old were similar, and therefore these groups were combined to result in two cohorts that were evenly sized by chance. Data pertaining to the age of dysphonia onset was unavailable for almost half of the patients in the study which limited its use as a metric for analysis. However, available data suggest that patients allocated to the first injection aged 60 years or younger cohort had significantly earlier disease onset compared to the older cohort.

The impact of age on BoNT‐A treatment for AdSD has previously been studied. In 2009, Vasconcelos et al. retrospectively reviewed treatment outcomes in 155 patients who had received more than five treatments of BoNT‐A for AdSD.^11^ In their analysis, patients were divided into two cohorts by age at the first treatment being less than, or greater than, 50 years of age. Neither the dose of BoNT‐A nor the length of benefit received were statistically significantly different.^11^ Subsequently, Lerner, et al. selected patients who had received more than 5 years of treatments of BoNT‐A for AdSD and divided by age <80 or >80 at the time of chart review.^12^ These inclusion criteria yielded 201 patients with no statistically significant difference between the cohorts in terms of mean dosing.^12^ These data differ from the findings of this study, where patients 60 years or younger at first injection tended to have a higher mean dose of BoNT‐A per treatment and a shorter mean length of benefit, but the mean maximal benefit achieved by treatment was similar. Posttreatment breathiness affected each cohort equally and posttreatment dysphagia did not statistically significantly differ but was approaching significance, with older patients possibly more susceptible to longer posttreatment dysphagia to liquids.

Vasconcelos et al. and Lerner et al. both studied the significance of sex along with age.^11^, ^12^ Vasconcelos et al. found similar mean treatment doses and mean length of benefit between men and women treated with BoNT‐A for AdSD. Lerner et al., however, found that the mean treatment dose received by women was significantly higher than men. Our analysis of sex resulted in expectedly uneven cohorts with a female predominance. The female cohort was older at first treatment and more likely to demonstrate coexistent vocal tremor. Replicating the findings of Lerner et al., the female cohort in this study received significantly higher doses of BoNT‐A. Other than differing dosing, our male and female cohorts were similar regarding the maximal benefit of treatment, length of benefit, and posttreatment breathiness and dysphagia.

This study represents the most highly powered publication to date addressing the impact of age and sex on the nature and efficacy of BoNT‐A treatment for AdSD. Mayo Clinic in Arizona has evaluated and treated over 700 patients diagnosed with AdSD for more than 25 years with a consistent treatment team. The practices employed in our institution are similar to those reported popular by practicing laryngologists, suggesting the results presented are widely applicable to the practices of physicians in the United States and patient outcomes could be expected to reflect those at other centers employing similar techniques.^7^

Significant limitations exist in this study. Data analyzed were collected prospectively over 25 years and analyzed retrospectively, presenting the opportunity for occult errors and biases to exist. The age of dysphonia onset was missing for nearly half of the patients which prevented its meaningful use in the study. Patients in the older cohort may have previously been treated in centers other than ours and attributed to the incorrect cohort despite this being considered in the analysis. The outcome metrics used were subjective and have not been validated. Importantly, data were collected at the patient's follow‐up appointment which introduced the possibility for significant recall bias. In both the analysis of age and sex, the cohorts had significant differences in their demographics and characteristics, including in the prevalence of vocal tremor. A retrospective study by Patel et al. found that patients with AdSD with vocal tremor achieved fewer good voice days following treatment with AdSD compared to patients with AdSD alone.^13^ These variations may bias our conclusions despite being controlled for using linear regression. Additional studies are required to provide the data necessary for a meaningful systematic review of the literature to address the roles of age and sex in the treatment of AdSD with BoNT‐A. Further study may enable meaningful individualized counseling and dosing based on a patient's sex and age at the initial consultation.

## CONCLUSIONS

Patients 60 years of age or younger at the first injection of BoNT‐A for the treatment of AdSD tended to have a higher mean dose of BoNT‐A per treatment compared to patients older than 60 years at the first injection. This younger cohort experienced a shorter mean length of benefit but the mean maximal benefit achieved by BoNT‐A treatment was similar. Females in this study received significantly higher doses of BoNT‐A. Other than differing dosing, our male and female cohorts were similar regarding the maximal benefit of treatment, length of benefit, and posttreatment breathiness and dysphagia.

## AUTHOR CONTRIBUTIONS

Richard Heyes contributed to the study design and conception, data collection, analysis, drafting, and approval of the final manuscript. Charles H. Adler contributed to the study design and conception, data collection, analysis, and approval of the final manuscript. Nan Zhang contributed to the study design, data analysis, and approval of the final manuscript. David G. Lott contributed to the study design and conception, and approval of the final manuscript. Stephen F. Bansberg contributed to the study design and conception, data collection, analysis, drafting, and approval of the final manuscript.

## CONFLICT OF INTEREST

The authors declare no conflict of interest.

## ETHICS STATEMENT

The above manuscript is the authors' own original work, which has not been previously published and is not in submission elsewhere. The paper reflects the authors' own research and analysis in a truthful and complete manner and properly credits the meaningful contributions of coauthors and coresearchers.

## Data Availability

Data are available on request from the authors.
